# 

**Published:** 1890-11-15

**Authors:** 


### NOTICE TO CORRESPONDENTS.

All MS., Letters, Books for Review, and other matters intended for the Editor should be addressed The Editor, The Lodge, Porchester Square, London, W.

The Editor cannot undertake to return rejected M.S., even when accompanied by stamped directed envelope.

**Notice to Advertisers and Subscribers.**

All Advertisements, Orders for Copies of The Hospital, and Business Communications should be addressed to The Publisher, *not to the Editor*, The Hospital, 140, Strand, W.C.

**Bound Volumes of "The Hospital."**

Vol. VI., containing full page portraits of T.R.H. the Prince and Princess of Wales, and Miss Florence Nightingale, is still obtainable.
Vol. VII. and all the earlier volumes are also for sale.
Orders will be received for Vol. VIII., 4s. post free. Cases for binding may be had of the Publisher, at 1s. 6d. each.

**To Residents, Secretaries, and Matrons.**

The Editor will be glad to have the Regulations and each Report as issued by Asylums, Hospitals, Convalescent and Nursing Institutions, as well as those of other Charities, and an early copy in duplicate of all Appeals and Festival Notices, etc., addressed to *The Editor, The Lodge, Porchester Square, London, W.* Any superintendent, secretary, matron, or other chief official who regularly sends such information may be registered, on application to the Publisher, to receive free of cost a cover for binding The Hospital in half-yearly volumes.

### BURDETT'S HOSPITAL ANNUAL FOR 1890

May be obtained of the Publisher, The Hospital Offices, 140, Strand, W.C., price 2s. 6d. limp boards, or 3s. 6d. cloth. All orders must be accompanied by a remittance.

### SCRAPS AND GLEANINGS.

Two warders have been murdered in France by a lunatic.

Liverpool issues a special appeal for a Maintenance Fund for the new infirmary.

A child, aged 4, died from alcoholic coma on board the *Umbria* during her last voyage.

Dartford needs a Cottage Hospital, but the funds are not forthcoming wherewith to build it.

The *St. James's Gazette* on October 28th commenced a series of papers on "At the Hospital."

Birmingham kept Hospital Sunday not only in the churches, but at the Town Hall and Grand Theatre.

Brighton Hospital Sunday Fund exceeds £1,000. At St. Margaret's Church the collection amounted to £110.

The Queen Victoria Convalescent Home, Belfast, which the Countess of Shaftesbury lately opened, will hold 12 beds.

There are over 2,000 patients in the fever hospitals just now, against 1,450 at the same time last year, and 1,000 in 1888.

At Harrow the winter concerts in aid of the Cottage Hospital have commenced. Other villages might take note; the concerts cheer the people when they are well, and provide them with comforts when they are sick.

The governor of Devon and Exeter Hospital had only one candidate for the post of physician before them on October 24th—Mr. William Gordon—so the appointment was promptly made.

Dr. Vichersheimer, of Berlin, has invented a new preserving fluid, which was injected into the bodies of the late Emperor William and Empress Augusta. The fluid is now being used to preserve meat.

The Secretary of Glasgow Western Infirmary pleads for plants and shrubs for the new winter garden for patients and nurses. What an advantage it would be if every hospital had its well kept winter garden!

Manchester Royal Infirmary has refused the offer of the Corporation to build them a new mortuary on the understanding that it should be of a public character. It was feared that unsanitary results might ensue from bringing more dead bodies about the place.

The Hospital for Sick Children, Great Ormond Street, has rescinded its rule that a medical officer should not hold office more than fifteen years. This was done on purpose to retain the services of Dr. Cheadle, who has ever been a good and generous friend of the institution.

Doncaster Infirmary admitted 74 in-patients last year, and treated 2,251 out-patients. The report is in every way satisfactory, though the Committee regret the loss by death of three of their patrons during the year.

### The Student of Medicine.

[All communications for this Supplement should be addressed to The Editor of The Hospital, at 140, Strand, with the words, "Students' Column," written in the left hand top corner of the envelope.]

### WOMEN STUDENTS AND MEDICAL EXAMINATIONS.

A long letter appeared in the *Times* of November 6th, from Mr. Thomas Case, of Oxford, with regard to the proposed statute for admitting women to the medical examinations. He certainly brings the full force of his arguments to bear on the subject, and we must admit that his opinions on the whole question of scientific and medical education are entitled to consideration. There are many points in his argument; we quote some of them. "It is not only unnecessary, but positively improper to initiate, educate, or examine young women in the essentially delicate subject of disease and medicine surrounded by young men in a university of men." Also that the statute would induce young women to study medicine in Oxford together with young men. The learned Tutor of Corpus then goes on to say that he has read the regulations of the Board of the Faculty of Medicine, and has glanced at the examination papers, and also finds to his horror that there is no statute which prevents the Regius Professor of Medicine from admitting women to his lectures. He proceeds to write: "At present a high moral tone prevails in the university; it would therefore be a matter of profound regret to Oxford and to English parents if, to use the expression of Canon Liddon, morals were seriously imperilled, as they certainly would be if there were young women in Oxford, and studying for medical examination the structures and the functions, the diseases and the cures, of the most secret organs of the human frame together with young men in a university of men." A letter of this nature having appeared in the leading daily paper, has attracted considerable attention. It is clothed in terms of gross insinuation not only to the class to whom it refers, but to the profession as a whole, and students in particular. It is a great pity that Mr. Case takes so much to heart what he, with his professional bias, considers the "morals" of the students. We might suggest to him that immorality comes from ignorance, and not from knowledge of Nature's laws, and that to the pure (physiologist) all things are pure. Also that the most secret organs of the human frame, those which as yet have remained a mystery to the investigators of man, are those which form the physical basis of what we call the "organ of the mind," and that the subject of psychology does not form a part of the medical examinations at his university."

MEN OF THE FUTURE.

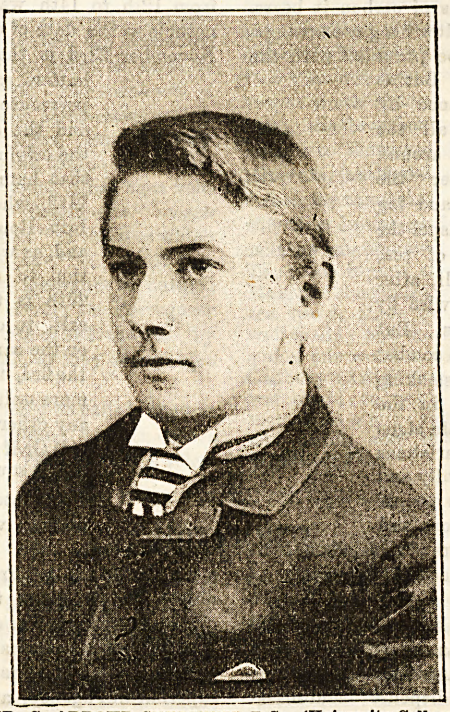

MR. G. ARBOUR STEPHENS, B.Sc. (University College), First-Class Honours in Materia Medica in Inter.B.M.B. London University.

### MEN OF THE FUTURE.

The portrait we give this week is that of Mr. G. Arbour Stephens, who was educated, first at the Grammar School at Cardigan, and then at the Independent College, Taunton; after this he went to University College of Wales, Aberystwyth, for three years, and from here matriculated, in 1886, at the London University, doing the Intermediate Scientific Examination the following year. In 1888 he took his B.Sc. with honours in Zoology, and entered University College Hospital the following October, passing the Intermediate M.B. Examination with First-class Honours in Materia Medica. We can but wish him equal success in the future.

### NOTES FROM THE SCHOOLS.

### EDINBURGH.

The University sustained its first defeat this season on Saturday, 8th, in the fourth round of the Scottish Cup (Association) against the Queen's Park team. The weather was wretched, and as it had been raining since noon, the ground was dreadful. The scoring was Queen's Park seven goals and University nil although it was several times very near scoring.

The Athletic Club held its bi-annual meeting on Friday, October 31st, Sir William Muir, Principal of the University, in the chair. Of course Professor Annandale was there—no University Athletic Club meeting would be complete without him. The cricket, lawn tennis, and cycling sections had a very successful season. The University did not do as well as it might have in the contest against the United Hospitals last July. There was great difficulty in getting a team. In the Scottish championships the University did very well. The subject of getting a new field nearer home was entered into, and there seemed to be a prospect of it being done.

The signs of the approaching Rectorial elections are very evident. The Conservative Association has large placards posted up. So has the Liberal Association. Two new papers, "The Q.C.," and "The Lord Rector," have been started by the Liberal and Conservative Associations respectively. "The Student" gave a portrait of Sir Charles Russell last week, and promises one of Mr. Goschen for this week.

The concert on the 8th, given by Madame Fanny Moody and Mr. Charles Manners, was a great success. It was in aid of the funds of the Union, and held in the debating hall of that building. Master Darby Pile, the Cornish boy pianist, eleven years old, began the concert with Weber's Rondo Brilliante in E flat. Mr. Charles Manners was loudly applauded for his song, "The Diver." Miss Dorothy Foster was encored for "The Better Land," so was Mr. Ellison for "My Queen"; both responded by giving "Venetian Love Song" and "The Distant Shore" respectively. "Scenes that are Brightest," from "Maritana," by Madame Fanny Moody, was enthusiastically encored. Miss Dorothy Foster was presented with a bouquet by Mr. A. Stodart Walker at the end of her song, "In the Chimney Corner." Madame Moody got her third encore for "Do as they do in England." "Home, Sweet Home," was given. Master Darby Pile's Rubinstein's melody in F, and the trio "S. Naviganti," by Madame Moody and Messrs. Charles Manners and Ellison closed the concert. Madame Moody's carriage was dragged to her hotel by students, the horses having been removed.

### STUDENTS' MEDICAL SOCIETIES.

### Middlesex Hospital.

On Thursday, October 30th, the society's prizes were presented to the successful competitors, as follows: Mr. J. W. Williams, for the best paper; to Mr. Stanley Thomas, for the best pathological specimens; and to Mr. K. Lawson, for the best histological collection. Mr. Spurr then read a paper on "Poisons and Poisoning." In his opening remarks he referred to the dread with which poison was regarded in the past ages, when ignorance, superstition, and credulity rendered wholesale poisoning a comparatively safe proceeding, and when, in consequence, it played an important part in the arrangement of domestic difficulties and the furtherance of political objects. He pointed out the fact that, owing to their not being allowed to make post-mortem examinations, the ancients possessed but little knowledge of anatomy and still less of pathology, and that the only diagnostic points in death from poisoning were great lividity and rapid decomposition of the corpse. Mr. Spurr then alluded to the bands of professional poisoners who arose during the Middle Ages in France and Italy, and to the poisons most in vogue amongst them. He showed how modern toxicology was distinguished from the poison lore of olden times by its greater extent, by its exactness, by the laborious compilation and verification of facts, and by the application of various instruments of precision, both at the bedside and in the laboratory. The discovery of vegetable alkaloids, and more recently of ptomaines, the application of tests for almost every known poison, the recognition of the relation between chemical composition and toxic activity, and the establishment of almost perfect antagonism between certain vegetable poisons, by the knowledge of which one powerful alkaloid is given to counteract the effects of another equally powerful, all these were referred to as amongst the

**Student's Medical Societies—*continued*.**

greatest triumphs of modern toxicology. After summarising the various theories as to how poisons act upon the system, Mr. Spurr concluded his paper by discussing the general principles of treatment of cases of poisoning. The president (Mr. A. Pearce Gould) alluding to the recent separation of the actual poison from micro-organisms by Dr. Martin, thought that poisoning might soon become a "fine art," that the murderer of the future might profit by recent discoveries and advances in toxicology. Mr. Fardon spoke of the manifest decrease in the number of cases of poisoning admitted year by year into the Hospital, and gave some excellent advice as to the general method of treatment to be adopted. The secretary thought that the various effects of poisons should be demonstrated to students while at the hospitals, that the sacrifice of the life of a few animals would be worth the increase of practical knowledge thus gained. Mr. Leopold Hudson referred to the prehistoric poison "Morion," derived from the "Autumn Mandrake." He explained how this poison used to be given to those who were crucified, as it had the effect of producing a coma undistinguishable from death itself. In this way many a man was laid to rest in a sepulchre hewn out of the rock, the coldness of his surroundings gradually brought him round again, and so he escaped death. When this trickery was found out, the method of breaking the legs of those who were crucified was instituted. Mr. Hudson then related how Dr. B. W. Richardson had imported the "Mandrake" from Athens, how it is growing now in Kew Gardens, and how he has extracted the poison and administered it to pigeons, which were thrown away for dead, but were found to be alive and well next morning.

**Guy's Hospital Pupils' Physical Society.**

A clinical evening was held on Saturday, November 1st, when the following cases were shown and discussed: Exophthalmic Goitre, by Mr. W. T. B. Donelly; Peripheral Neuritis, by Mr. A. M. Daldy; Obscure Abdominal Tumour, by Mr. F. M. Turner; Aphasia, by Mr. T. R. Taylor; Chronic Gout, by Mr. H. E. Durham; Sarcomatous Glands in Neck, and Epithelioma of the Tongue, by Mr. A. J. Sharp. The following pathological specimens were also shown: Sarcoma of Mediastinum, by Mr. W. D. Loveday; Tubercular Epididymitis, by Mr. C. M. Greenway; Membraneous Laryngitis in Measles, by Mr. J. M. Gill; Pneumonia and Pericarditis, by Mr. A. E. Durham; Retroperitoneal Cyst, by Mr. J. M. Gill; Coccidia in connection with Sarcoma of the Heart, by Mr. H. E. Durham.

### SCHOLARSHIPS AND PRIZES.

**The London Hospital Medical College.**

The Hutchinson prize, of the value of £35, given triennially, has been awarded to Mr. John Thomas, of the Infirmary, Cardiff.

**Adelaide Hospital, Dublin.**

The following prizes have been awarded for the past session Hudson scholarship, gold medal and £30, Thomas E. Gordon; Hudson prize, silver medal and £10, T. W. Fullerton; medical prize, J. H. Farmer; surgical prize, J. G. Boon and J. H. Farmer (equal).

**St. George's Hospital.**

The following entrance scholarships have been recently awarded at St. George's Hospital: The £125 scholarship, open to the sons of medical men, to Mr. Charles R. Watson; the £50 to Mr. C. H. Nicholls; and the £65 scholarship, open to members of the Universities of Oxford and Cambridge, to Mr. William Hawkins Wilson.

**Cambridge, November 3.**

At St. John's College the annual election to Fellowships was held to-day, when the following were chosen: 1. Lewis Robert Shore, M.A., First Class Natural Sciences Tripos, Part I., June, 1884; First Class Natural Sciences Tripos, Part II., June, 1885, for Physiology. 2. Ernest Hanbury Hankin, B.A., First Class Natural Sciences Tripos, Part I., June, 1888; First Class Natural Sciences Tripos, Part II., June, 1889, for Physiology.

### PASS LISTS.

**Royal College of Physicians of London.**

The following gentlemen having passed the necessary examinations, and having conformed to the bye-laws and regulations, were at the last meeting of the College admitted licentiates, viz.: Messrs. F. H. Alderson, student of Durham University and Middlesex Hospital; W. E. Almas, Toronto University; R. W. Anderson, University College; H. Andrew, St. Thomas's; A. E. Ash, Royal Infirmary, Manchester; W. B. Bale, Royal Infirmary, Manchester; W. K. Bell, Charing Cross; G. R. Bickerstaff, St. Mary's; A. H. Bindloss, St. Mary's and Cambridge University; T. G. Brodie, King's College; F. G. Bushnell, University College; H. A. Caley, St. Mary's; D. M'D. L. Campbell, St. Mary's; H. B. Cardew, St. Bartholomew's; A. Carney, St. Bartholomew's;

**Pass Lists**—(*continued*).

H. J. Carstairs, St. Thomas's; F. J. Charlton, University College; C. Coles, St. Bartholomew's; R. C. M. Colvin-Smith, St. George's and Cambridge University; C. D. Cooper, University College; J. M. Crocker, Yorkshire College and the General Infirmary, Leeds; A. Crompton, St. Bartholomew's; L. W. Dent, King's College; C. W. Emlyn, St. Bartholomew's; F. W. Farr, Guy's; C. H. Fowler, St. Bartholomew's; Anghel Gaster, Bucharest University; B. Goddard, St. Thomas's; A. M. Gossage, Oxford University and Westminster; W. S. Griffith, St. Thomas's; J. F. Gummow, Charing Cross; J. B. Hall, Cambridge University; R. Hamilton, Manchester Royal Infirmary; J. R. Harper, St. Thomas's; F. Harris, Liverpool Royal Infirmary; C. S. Hawkes, London; T. J. Henning, Galway and St. Bartholomew's; W. H. Henshaw, Manchester Royal Infirmary; W. Hichens, London; R. H. E. G. Holt, St. Mary's; W. Horsfield, Manchester Royal Infirmary; G. Y. Cobb-Hunter, St. George's; H. S. Jackson, Bristol Royal Infirmary; H. W. John, Guy's; D. J. Jones, Charing Cross; W. J. C. Keats, St. Bartholomew's; C. I. Kirton, London; G. H. Knapp, Guy's; C. P. Le Quesne, St. Bartholomew's; G. D. B. Levick, Middlesex; R. M. Littler, Manchester Royal Infirmary; G. Lys, London; H. F. Mantell, St. Mary's; H. Mason, General Hospital, Birmingham; S. F. Mawson, Manchester Royal Infirmary; H. F. Mole, Bristol Royal Infirmary; J. E. Molson, Middlesex; M. S. F. Monier-Williams, St. George's; E. M. Morphew, University College; W. B. Morton, University College; H. A. de B. Nelson, St. Bartholomew's; W. T. Ohlmus, Ceylon Medical College; E. C. Osborne, Westminster; J. Panting, Cambridge University and Middlesex; T. L. Pennell, University College; C. S. Pethick; St. Bartholomew's; H. M'D. Phillpots, St. Mary's; H. W. Pooler, Birmingham General Hospital; F. B. Reilly, Charing Cross; G. L. H. Revill, Charing Cross; N. L. Richards, Guy's; R. W. Richards, St. Bartholomew's; L. Roberts St. Bartholomew's; E. C. Ryall, Westminster; A. W. R. Sayres, St. Thomas's; J. R. Scott, St. Thomas's: J. H. Shaw, Liverpool Royal Infirmary; W. Rushton Shortt, St. George's; H. A. Smith, Guy's; W. A. Stott, Leeds General Infirmary; R. Stuart, St. George's; H. Tempest, Leeds General Infirmary; H. J. Tizard, St. George's; W. F. Umney, St. Thomas's; C. M. Vernon, Bristol Royal Infirmary; L. Wainwright, London; R. M. H. Walford, St. George's; K. S. Walfridsson, London; W. B. Wedgwood, King's College; A. J. Williams, King's College; J. G. Wilson, London; G. M. Winter, St. Mary's; G. E. M. Wood, University College; P. Worley, Manchester Royal Infirmary; and Selim Zeidan, St. Thomas's.

### ATHLETICS.

### Football.

Association Rules.—On November 4th.

*St. Thomas's Hospital* lost to the *Casuals* yesterday at Wormwood Scrubs by four goals to one.

On November 8th.

*Warwick County v. Oxford University.*—In consequence of the wretched state of the weather there were very few people at the County Ground to witness this match. The home team were in great form, Pangbourne scoring for them at the outset. After some even play Rhodes equalised, and then the visitors had the best of it, Rhodes eventually adding a second goal. Just before half-time Hare equalised for the County, and upon change of ends the 'Varsity star was again in the ascendant, Wilson scoring their third goal. This proved to be their final point as the County outstayed them, and, playing with rare dash, they added three goals to their score, and when "time" was called Warwick County were left victorious by five goals to three.

*Cambridge University* beat *Northamptonshire*, at Wellingborough, before 3,000 spectators. The visitors pressed soon after start, and at half-time scored three goals to nil. In the second portion of play they again hotly attacked, and added three more goals. A few minutes before the close the home players scored twice in succession. The home men weak in front. *King's College Hospital* beat *Charing Cross Hospital (A)*, at Wormwood Scrubs, by four goals to one. *King's College Hospital* beat *Polytechnic Wanderers (A)*, at Wormwood Scrubs, by two goals to none. *Lewisham Park* beat *London Hospital (A)*, at Ladywell, by two goals and three tries to nil. *St. Bartholomew's Hospital* beat *Old Foresters*, at Snaresbrook, by five goals to nil. *Watford Rovers* drew with *St. Mary's Hospital*. The Rovers were weak; Fred Sargent and Welter Coles away. The Hospital fully represented. A fast and very even game was played. Darkness stopped game. *Guy's Hospital and University 2nd* resulted in the victory of the former with ten goals to four. *Old Salways met Charing Cross Hospital*, and defeated them by five to nil. *St. Bartholomew's Hospital* against *Erith* scored two goals to the latter's nil.

Rugby Rules—November 8.

*St. Thomas's Hospital v. Royal Military College.*—This match took place on Saturday in fine weather on the ground of the latter at Sandhurst before a fair gathering of onlookers. In the opening half, playing into the entrance goal with the wind in their teeth, the visiting team pressed their opponents somewhat severely. After a free kick for off-side, Fisher made a try for the visitors, but Ogilvie in both cases failed to establish a premier point. The Sandhurst team now played with greater vigour, but it was not till five minutes before half time that Guthrie-Smith was able to put on a goal for his side, Hely-Hutchinson making a try. In the second moiety the game became more even, although the visitors still held a slight advantage. Wingate, Irvine, M'Conaghey, Foote, Guthrie-Smith, Northcote, Fisher, Ord, and Haward were very conspicuous for their respective sides. Northcote made two tries for the visitors after some brilliant runs, one of which Ogilvie managed to convert into a goal. In the end victory rested with the visitors by one goal and three tries to one goal one try. Teams: R.M.C., Sandhurst—Cattell (back), Hely-Hutchinson, Hall, and Johnson

**Athletics—*continued.***

(three-quarter backs), Fagan and Wingate (half-backs), Barnett, Carter, Norrington, Knatchbull. Irvine, M'Conaghey, Foote, Guthrie-Smith, and Brackenburg. St. Thomas's Hospital—A. Wishaw (back), E. Bromet, J. Ogilvie, P. V. T. Grahame (three-quarter backs), P. Northcote, J. Fisher (half-backs), W. Jackson, H. Greaves, F. Goodhue, R. W. Ord, P. O. Gabbett, W. Ashford, J. Carver, F. J. G. Turner, and H. Haward.

*Oxford University* beat *London Scottish*, in the Parks, at Oxford, in the presence of 3,000 spectators. Lindsey kicked off for the Scots at half-past three, the exchanges leaving the ball in neutral ground. Some capital exchanges between Easterbrook, Anderson, Lindsey, and Campbell carried the fight into the home twenty-five, and Riddle nearly got over, the Oxonians being forced to touch down. Resuming, a rush was made by the 'Varsity forwards headed by Rice, Evans, and Percival, and passing by De Winton, Percival and Kay caused the Scots' territory to be invaded. Lindsey relieved, but Cochran returned. Anderson came to the rescue with a long kick to the centre, where Cockran got possession, and dropped a magnificent goal for the Dark Blues. After the kick-out, a rush by the 'Varsity forwards, in which Kay, Cookson, and J. H. Wilson were conspicuous, was splendidly stopped by Anderson, and play for a time was carried on in the centre. Coventry at length got possession out of a scrimmage, and made a capital run, and on being collared, transferred to Kay, who gained a try for the Collegians which Cochran failed to improve upon; and half-time was taken with the score: Oxford University, one goal, one try; London Scottish, nil. With change of ends, the Dark Blues were seen to great advantage, and they completely penned the Scots, the second half of the game being entirely carried on in their twenty-five, the ball never once being taken into the 'Varsity half. Parkin and Percival gained further tries for Oxford, from the last of which S. E. Wilson scored the major point, and when the whistle finally sounded the Scots left the field decisively beaten by two goals and two tries to nil. Oxford University: P. Cochran (Oriel) (back), C. J. N. Fleming (Queen's) captain), P. R. Clauss (Keble), and W. H. Parkin (Brasenose) (three-quarter-backs), R. G. Coventry (New) and R. F. C. de Winton (Exeter) (half-backs), W. Rice Evans (Jesus), J. H. G. Wilson (Queen's), A. R. Kay (Oriel), A. M. Paterson (New), L. J. Percival (Trinity), G. F. Cookson (Lincoln), E. Bonham-Carter (New), R. W. Hunt (Corpus), S. E. Wilson (Trinity). London Scottish: H. J. S. Gedge (back), G. C. Lindsay, G. T. Campbell and J. G. Paterson (three-quarter backs), D. E. Sanderson and R. F. Easterbrook (half backs), J. H. Heddernick, T. R. Riddell, A. Scott, N. F. Henderson, A. H. Grant, A. Harvey, J. D. Macdonald, N. Ferguson, and F. J. L. Ogilvie.

*Cambridge University* drew with *Harlequins*, at Cambridge, the 'Varsity scoring a goal, obtained from a try, and a dropped goal, while the Harlequins got a goal from a free kick, and two tries. For the visitors F. De L. Solbe was instrumental in securing the first try, while F. B. Hannen also secured a try, and just before the whistle sounded A. B. Cipriani made his mark, and G. B. James obtained a goal from the free kick awarded. For Cambridge, W. Wotherspoon dropped a goal, and converted another obtained from a try by A. B. Fforde.

*Old Paulines* beat *Westminster Hospital*, at Raynes Park by three goals and one try to one goal. *St. George's Hospital* beat *Tonbridge School* by two goals and two tries to one goal. *Saracens (Second)* beat *Middlesex Hospital (Second)*, at Walthamstow. Saracens played a very superior game; their forwards dribbled splendidly, and the passing behind the scrum quite nonplussed their opponents. Penstone scored after good passing by Lawrence. Tibbs, and others, and Fulford obtained a good try. The result was a win for Saracens by two tries and three minors to nil. *St. Bartholomew's Hospital* beat *Erith* by two goals to one. *University College Hospital (Second)* beat *Wickham Park (Third)* by three goals to nil. Griffiths placed two goals, and Mills dropped the third. *Walford Rovers* drew with *St. Mary's Hospital*—no score. *Warwick County* beat *Oxford University*, at County Grounds, Birmingham, by five goals to one. *Wasps* beat *London Hospital*, at Putney, by two goals (one from a try and one from a penalty kick) to one (dropped goal), and a try for Wasps by Leetham; goal placed by Kidd. *Kensington* and *Guy's Hospital*, at Wood Lane, drawn (one try each). *Lewisham Park* beat *London Hospital*, at Ladywell, by two goals and three tries to nil. *Barnes* beat *St. Thomas's Hospital* by three goals to nil. *Middlesex Hospital* beat *Civil Service* by a try to nil. *Thurlow Park* beat *St. Mary's Hospital*, at Streatham, by one goal and three tries to nil. *St. Thomas's Hospital (Second)* beat *Guy's (Second)*, at Lambeth, by three goals and four tries to nil. *Saracens (Second)* beat *Middlesex Hospital (Second)*, at Walthamstow, by two tries to nil. *Wickham Park* beat *St. Bartholomew's Hospital*, at Lee, by one goal to nil. Swift scored during the first half. Wright kicking a goal. *St. Bartholomew's Hospital* ran away with *Marlborough Nomads*, who sustained defeat by four goals two tries to one try.

**United Hospitals H. and H.**

The meet of this club on Nov. 8th took place at the headquarters at Raynes Park. The course lay first past the station, on to the short distance course, then bearing to the left to Norbiton, then, still to the left, the "water jump" was crossed twice, and from here into Old Maldon, finishing up with the challenge cup course. There was a fast run in on the road for the last mile. The result was: (1), C. H. Munro (Guy's); (2), A. N. Wilde (Bart.'s); (3), F. E. Easton (Mary's).

**EVENTS FOR THE MONTH OF NOVEMBER.**

[Items intended for this column must reach the office, 140, Strand, W.C., not later than first post on Monday mornings.]

**November Meetings and Athletics.**

For list of Students' Medical Societies' Meetings and Football Matches during November, see THE HOSPITAL for Nov. 1st, p. 84.

### GENERAL CHARITIES.

[This Column is devoted to an account of work of charitable institutions other than Medical Charities. The Editor will be glad to receive reports or news of work at 140, Strand. *Philanthropy* should be plainly written on the envelope.]

At the half-yearly General Court of **THE GOVERNESSS' BENEVOLENT INSTITUTION** held on Friday, the 7th inst., one candidate was elected for the Asylum, seven were elected for annuities of £25 each, one for the Kihavack annuity of £32, and one for the Fryer annuity of £34 2s. 6d. The six highest unsuccessful candidates received £10 each as a donation.

An urgent appeal is made by the Board of Management of **THE SHIPWRECK D FISHERMEN AND MARINERS' ROYAL BENEVOLENT SOCIETY** for funds in special aid of sufferers through the recen' gales which have been especially destructive on the South-west coasts of the United Kingdom. The urgent necessities of the hipwrecked sailors themselves as well as of the destit te widows and orphans require more prompt aid than those of many other classes Donations will be gratefully acknowledged by the Secretary, Mr. W. R. Buck, Sailors' Home Chambers, Dock Street, E.

The operations of **THE LONDON SAMARITAN SOCIETY** at Homerton Mission are somewhat varied. During the winter months the society extends charitable relief in food and clothing, for which the poorer districts of London provide a never ending number of needy applicants. As the greater part of the society's income is made up of small sums, they are grateful for any presents of clothing, boots, blankets, sheets, vegetables, fruit, meat, poultry, or game. Naturally the harder the winter the more numerous become the applicants for assistance. In numerous cases free dinners are supplied to children and occasionally Sunday morning breakfasts. No doubt the society in this department of its operations must frequently be imposed upon however carefully the work is carried on, but it is quite impossible for the most carefully conducted charity to do any good without doing a certain amount of pauperising. It is seldom or possible to get at the whole truth. Presumably recognising this fact, the society has discontinued giving Sunday morning free breakfasts on as large a scale as formerly, experi nce having taught them that the only way in which imposition can be avo ded with any approach to success is the adoption of a system of relief which reduces the risk to a minimum. The society now has arrangements with nearly six hundred coal merchant, bakers, provision dealers, and cocoa houses by which they will recognise the relief tickets which they issue. These tickets are issued, on application for them, to district and other reliable visitors among the deserving poor, and are distributed according to the peculiar necessities of those whom they visit. Possession of the tickets entitles the holder, on presentation, to loaves of bread of two or four pounds, a packet of grocery or provisions, a meal of bread and cocoa, a hundredweight or half-hundredweight of coals, dinners for the children, or lodgings for the night, as the case may be. Thus without money gifts, which are such potent destroyers of independence and self-reliance, the society ministers immediate relief to those who they have found on inquiry to be in need and deserving of help. So many of the poorest of the poor in the East-end of London, after being taken ill and being consequently out of work, sometimes for a long time are unable to get fresh employment owing to their inability to present a respectable appearance. In such cases the gift of a pair of boots or some decent clothes helps the willing to help themselves. At Homerton there is a clothing department, where hands are constantly employed in making, mending, altering or fitting the various articles of clothing sent for distribution. Occasionally the society has been enabled not only to supply its own immediate neighbourhood, but to send parcels to local workers in other districts for the purpose of distribution. The cases which come before the council are generally those of sad destitution if not of absolute starvation. The pallid and old-before-their-time faces of many children tell their own tale, how for weeks they have had but one meal a day, and that consisting of dry bread and weak tea. Such cases as these appeal, or should appeal, to all who are able to make the lives of those around them brighter and purer and happier. Many who cannot afford donations can, without any drain on their resources, help with gifts of old clothing, which will help during the coming winter to bring back vigour to many a frame weakened through illness or daily toil in unhealthy surroundings. The happiness given and the good thus done compared with the cost of giving are immense. In addition to this mission work in the East of London, the society prov des convalescent homes at Sandgate and Bournemouth for men, women, and children from all parts of the kingdom. A subscriber's letter is necessary for admission, and those who can afford it are expected to pay five shillings a-week for the three weeks they are allowed the benefits of the home. There is, however, a fund for providing free admissions. Donations to this fund, as well as to the fund for enlarging the home at Sandgate, and any gifts of provisions or clothing for the general relief work, will be gratefully acknowledged by Mr. J. J. Jones, 98, High Street, Homerton, E.

